# *Otof* gene transfer in DFNB9 mice carrying human founder non-truncating alleles

**DOI:** 10.1016/j.gendis.2025.101590

**Published:** 2025-03-06

**Authors:** Yehree Kim, Yoojin Chung, Ju Ang Kim, Kyu Hee Han, Kwon Woo Kang, Ngoc-Trinh Tran, Min Young Kim, Eunyoung Yi, Sangyong Jung, Bong Jik Kim, Quynh-Anh Artinian, Seth D. Koehler, Ning Pan, Tyler M. Gibson, Lars Becker, Joseph W. Goodliffe, Molly Kalker, Madeline Barnes, Luke A. Shaheen, Meghan C. Drummond, Vassili Valayannopoulos, Byung Yoon Choi

**Affiliations:** a Department of Otorhinolaryngology, Seoul National University Bundang Hospital, Seoul National University College of Medicine, Seongnam 13620, South Korea; b Decibel Therapeutics, Inc., Boston, MA 02215, United States; c Regeneron Pharmaceuticals, Inc., Tarrytown, NY 10591, United States; d Department of Otorhinolaryngology, National Medical Center, Seoul 04564, South Korea; e College of Pharmacy and Natural Medicine Research Institute, Mokpo National University, Muan 58554, South Korea; f Department of Medical Science, College of Medicine, CHA University, Seongnam 13488, South Korea; g Department of Otorhinolaryngology, Chungnam National University School of Medicine, Chungnam National University Sejong Hospital, Sejong 30099, South Korea; h Brain Research Institute, Chungnam National University College of Medicine, Daejeon 35015, South Korea; i Eaton-Peabody Laboratory, Massachusetts Eye and Ear Infirmary, Boston, MA 02114, United States

Gene therapy has shown promise for treating sensorineural hearing loss, supported by numerous successful preclinical studies. From the perspective of translation to humans, researchers have focused more on the genetic causes of profound sensorineural hearing loss, where the sensory epithelium remains viable and intact for a considerable time after birth in humans. A key human deafness gene that best fits such a context is *OTOF* (GenBank AF183185.1), of which protein products, otoferlin, is essential for synaptic exocytosis and vesicle replenishment at the inner hair cell level in the cochlea.^1^ Several preclinical studies where *Otof* cDNA was transferred into the cochlea of adult *Otof* null allele mice, including both *Otof*^Δ/Δ^ and *Otof*^*p.Q939*^*∗*^*/p.Q939*^*∗*, successfully achieved near-normal thresholds,^2^ and furthermore, relatively successful human clinical trials of gene therapy have been conducted for DFNB9 (OMIM 60381) patients showing auditory neuropathy.^3^^,^^4^ One important consideration is that, to date, there has not been a single case in any preclinical study or human clinical trial where both alleles of *OTOF*/*Otof* were non-truncating. However, many human patients with DFNB9 carry non-truncating missense variants in *OTOF*, indicating that their inner hair cells express some mutant RNA/proteins. This raises questions about how these mutant RNA/proteins might interact with exogenous transgene products, affect the function of newly expressed otoferlin, and influence the outcome of gene therapy. To address this, research on wild-type *Otof* cDNA transfer in mouse models with two copies of common founder missense variants, like p.Arg1939Glu (p.R1939Q), prevalent in Korean and Japanese populations,^5^ is essential.

We generated *Otof*^*p.R1934Q/p.R1934Q*^ mice harbouring a variant (*Otof* p.R1934Q) corresponding to a founder human variant (p.R1939Q) in Korean and Japanese DFNB9 patients. Among 81 F0 pups born to the injected egg (Fig. 1A), two carried the mutagenized knock-in allele (Fig. S1). In F2 lines, *Otof*^*+/+*^ and *Otof*^*+/p.R1934Q*^ pups showed normal auditory brainstem response (ABR) thresholds, while *Otof*^*p.R1934Q/p.R1934Q*^ mice had minimal responses, even at 100 dB at 1–3 months (Fig. 1B; Fig. S4). These mice retained DPOAE responses similar to *Otof*^*+/+*^ controls at 5 weeks (Fig. 1C), mimicking the human DFNB9 phenotype caused by p.R1934Q. Immunohistochemistry assays quantified p.R1934Q *Otof* mRNA and protein across genotypes (Fig. 1D). Normalized fluorescence intensity revealed that otoferlin decreased to 26% and Vglut3 to 65% of *Otof*^*+/+*^ controls (Fig. 1E). This decrease was more prominent in the inner hair cell's basal part than the apical portion (Fig. 1F), suggesting an apical shift of remaining mutant otoferlin (Fig. 1G and H). *Otof* mRNA levels, measured by RNAscope and real-time PCR, showed no significant difference between *Otof*^*+/+*^ and *Otof*^*p.R1934Q/p.R1934Q*^ (Fig. S2). To determine whether the apically shifted, unstable mutant otoferlin impacted hair cell survival, hair cells in *Otof*^*+/+*^ and *Otof*^*p.R1934Q/p.R1934Q*^ mice were stained and counted (Fig. 1I; Fig. S3). No significant difference in inner hair cell or outer hair cell numbers was found between 4-month-old *Otof*^*+/+*^ and *Otof*^*p.R1934Q/p.R1934Q*^ mice (Fig. 1J), suggesting hair cell survival was not significantly affected by *Otof* p.R1934Q. DPOAE tests on *Otof*^*p.R1934Q/p.R1934Q*^, *Otof*^*+/p.R1934Q*^, and *Otof*^*+/+*^ mice examined DPOAE maintenance over time (*n* = 6, 12 ears for *Otof*^*p.R1934Q/p.R1934Q*^; *n* = 5, 10 ears for *Otof*^*+/p.R1934Q*^; *n* = 5, 10 ears for *Otof*^*+/+*^ mice). DPOAEs were generally preserved in *Otof*^*p.R1934Q/p.R1934Q*^ mice except for 16–19 kHz responses at 2 months. Most otoacoustic emissions remained intact up to 5 months. However, from 6 months, significant decreases were seen at 6, 8, and 10 kHz compared with *Otof*^*+/p.R1934Q*^ and *Otof*^*+/+*^ mice (Fig. 1K).

**Figure 1:**
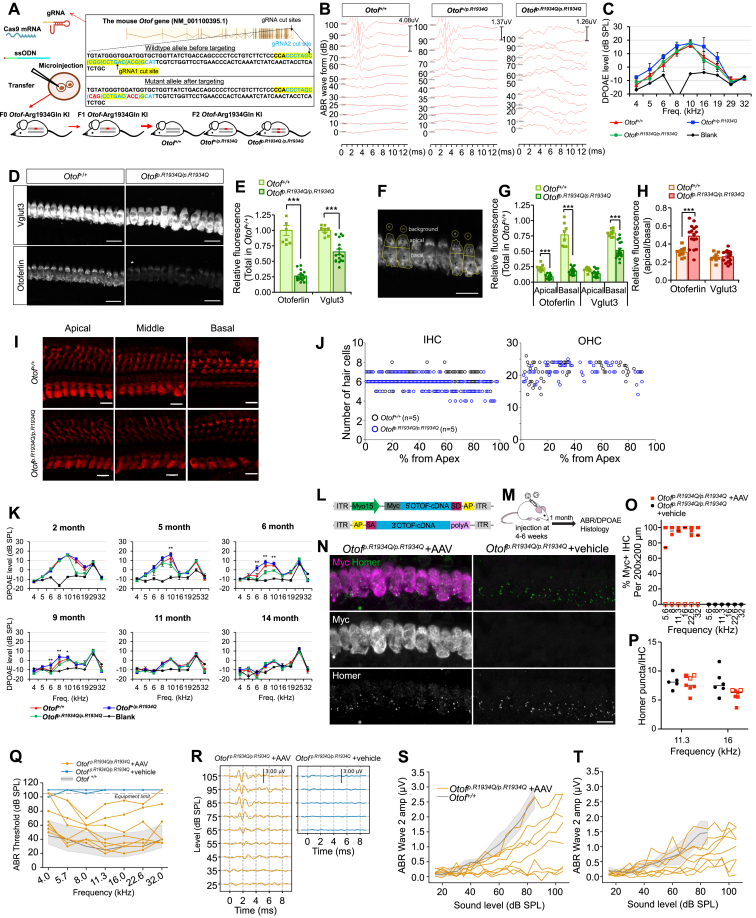
Audiological phenotype of *Otof*^*p.R1934Q/p.R1934Q*^ KI mice and gene transfer therapy for treatment of auditory neuropathy spectrum disorder due to the Otof p.R1934Q. **(A)** Schematic depiction of the targeting strategy for the generation of *Otof*^*p.R1934Q/p.R1934Q*^ KI mice. **(B)***Otof*^*p.R1934Q/p.R1934Q*^ mice did not show any meaningful ABR, while *Otof*^*+/+*^ and *Otof*^*+/p.R1934Q*^ mice still showed normal ABR threshold at 3 months of age. **(C)***Otof*^*p.R1934Q/p.R1934Q*^ mice exhibited comparable DPOAE responses to that of *Otof*^*+/+*^ and *Otof*^*+/p.R1934Q*^ mice at 5 weeks of age. **(D)** Z-projected confocal images of cochlear IHCs of indicated genotypes of *Otof*^*+/+*^ and *Otof*^*p.R1934Q/p.R1934Q*^ mice were analyzed for comparison of normalized fluorescence intensity of otoferlin and Vglut3. **(E)** The level of otoferlin and Vglut3 protein expression was significantly reduced in *Otof*^*p.R1934Q/p.R1934Q*^ mice. **(G)** The ratio of apical and basal protein levels. The ratios of otoferlin in *Otof*^*p.R1934Q/p.R1934Q*^ mice versus *Otof*^*+/+*^ controls were significantly decreased. The decreased level of otoferlin was more pronounced at the basal part of IHCs. **(H)** The ratio of apical/basal protein levels of otoferlin and Vglut3. The ratio of apical otoferlin in *Otof*^*p.R1934Q/p.R1934Q*^ mice was significantly increased compared with *Otof*^*+/+*^ controls, indicating that remaining otoferlin shifted apically. **(D**–**H)***Otof*^*+/+*^, 8 IHCs from *n* = 2; *Otof*^*p.R1934Q/p.R1934Q*^, 16 IHCs from *n* = 4; scale bar = 20 μm; the data were presented as mean ± standard error; two-way analysis of variance with Bonferroni posttest; *∗∗∗p* < 0.001. **(I)** Representative images of hair cells in apical (10%–20% from the apex), middle (45%–55% from the apex), and basal turns (75%–85% from the apex) from *Otof*^*+/+*^ and *Otof*^*p.R1934Q*^*/*^*p.R1934Q*^ mice. IHCs and OHCs were immunolabeled with anti-myosin 6 (red). Scale bar = 10 μm. **(J)** Numbers of hair cells in a segment spanning 1% of the whole cochlear length. No hair cell loss of either IHCs or OHCs is shown in *Otof*^*p.R1934Q/p.R1934Q*^ mice at 4 months of age. *n* = 5 for *Otof*^*+/+*^ and *Otof*^*p.R1934Q/p.R1934Q*^ each group. **(K)** DPOAEs over time from each DFNB9 genotype. At 2 months of age, OAE responses still existed except those from 16 to 19 kHz. A substantial portion of OAE responses from *Otof*^*p.R1934Q/p.R1934Q*^ mice was preserved until 5 months of age. The levels of the signals were L1 = 65 dB and L2 = 55 dB. The stars indicate significant differences between *Otof*^*p.R1934Q/p.R1934Q*^ and *Otof*^*+/+*^; one-way ANOVA with Tukey's multiple comparison test; ∗*p* < 0.05 and ∗∗*p* < 0.01. **(L)** Schematic of the dual hybrid vector system with m*Otof* coding sequences. **(M)** Dual vectors were injected through the round window in mature mice (4–6 weeks of age). **(N)** Confocal immunofluorescence imaging of IHCs in AAV- and vehicle-treated *Otof*^*p.R1934Q/p.R1934Q*^ mice with anti-Myc tag and Homer co-labeling. Scale bar = 10 μm. **(O)** Percentage of Myc^+^ IHCs across frequency regions. Most animals showed > 50% of IHCs with Myc signal. **(P)** Homer puncta count per IHC. The horizontal bars indicate the median, and the open symbols reflect treated animals without Myc-signal for (O) and (P). The open symbols indicate animals with no Myc-otoferlin detected. **(Q)** ABR thresholds of all individual animals treated with dual AAV vectors (*n* = 8) and vehicle (*n* = 4). All animals treated with AAV vectors showed detectable ABR thresholds, with most animals in normal hearing range (the grey shaded areas indicate the 90% interval of observed thresholds in normal hearing C57BL/6 mice; the black line indicates the mean). In contrast, animals treated with the vehicle only show no detectable ABRs below the equipment limit. **(R)** ABR at 4 weeks post-treatment in AAV- and vehicle-treated *Otof*^*p.R1934Q/p.R1934Q*^ mice. ABR waveforms in response to 16 kHz tone bursts were restored in AAV-treated mice, whereas vehicle-treated mice showed no detectable ABRs. **(S)** ABR wave I amplitude growth curves of individual animals. **(T)** ABR wave II amplitude growth curves of individual animals. The grey shaded area in panels (R–T) indicates the mean and standard deviation of wave amplitudes in *Otof*^*+/+*^ mice. Wave I amplitudes were smaller than those of normal hearing mice, whereas wave II amplitudes were comparable to those of normal hearing mice. The dashed lines indicate mice (*n* = 3) with no Myc-otoferlin detection. ABR threshold and amplitude recovery in these mice were poorer than the mice with robust expression. gRNA, guide RNA; KI, knock-in; mRNA, messenger RNA; m*Otof*, mouse otoferlin; ssODNs, single-stranded oligodeoxynucleotides; ABR, auditory brainstem response; DPOAE, distortion product otoacoustic emission; SPL, sound pressure level; IHC, inner hair cell; SE, standard error; Vglut3, vesicular glutamate transporter 3; OHC, outer hair cell; OAE, otoacoustic emission; AAV, adeno-associated virus.

A viral-*Otof* cDNA vector was created to rescue the phenotype of *Otof*^*p.R1934Q*/*p.R1934Q*^ mice through gene transfer therapy. The murine otoferlin cDNA was divided into a 5′ fragment (*Otof* NT) and a 3′ fragment (*Otof* CT), which were inserted into separate AAV vectors containing a recombinogenic bridging sequence. The AAV-*Otof* NT recombinant vector was designed to carry the mouse Myo15 promoter sequence, a Myc tag, and the 5′ part of the cDNA followed by a splice donor site. The AAV-*Otof* CT recombinant vector was constructed to include a splice acceptor site followed by the 3′ part of the cDNA (Fig. 1L).

These recombinant vectors were then packaged into AAV1 capsids for delivery. A single unilateral injection (2 μL) of the AAV-*Otof* NT plus AAV-*Otof* CT recombinant vector pair was administered to *Otof*^*p.R1934Q*/*p.R1934Q*^ mice aged 5 weeks through the left posterior semicircular canal (*n* = 8 for AAV; *n* = 4 for vehicle) (Fig. 1M). Four weeks after the injection of the recombinant vector pair, the sensory epithelium of the treated cochleae of *Otof*^*p.R1934Q*/*p.R1934Q*^ mice were micro-dissected and immunolabeled for anti-Myc and Myo7A to estimate the inner hair cell transduction rate (Fig. 1N). Anti-Myc labeling was detected in the inner hair cells of the five mice, which showed better ABR restoration. Up to 5 weeks of age, the otoacoustic emissions in *Otof*^*p.R1934Q*/*p.R1934Q*^ mice were perfectly preserved, identical to those in *Otof*^*+/+*^ mice. In these five mice, otoferlin expression was detected in more than 90% of the inner hair cells (mean ± standard deviation: 97% ± 5%; *n* = 5 cochleae; Fig. 1O). No Myc-signal was detected in three mice which showed less ABR restoration, presumably due to low levels of otoferlin expression. The cochlear wholemounts were co-immunolabeled with anti-Homer to evaluate the recovery of postsynaptic components after treatment. The number of Homer-positive puncta per inner hair cell of the AAV-injected group (mean ± standard deviation: 6.71% ± 1.42%) was not different from the vehicle-injected group (8.54% ± 1.52%) (Fig. 1P).

ABR recordings 4 weeks after the injection of dual Myc*-Otof* AAV demonstrated a substantial restoration of hearing thresholds (4, 5.7, 8, 11.3, 16, 22.6, and 32 kHz) in all of the treated mice (*n* = 8), but no restoration in the *Otof*^*p.R1934Q*/*p.R1934Q*^ mice receiving only vehicle (*n* = 4; Fig. 1R). The ABR thresholds of all treated mice showed restoration of cochlear function, with the majority in the normal hearing range (Fig. 1Q; Fig. S5). Out of eight mice, five showed high levels of Myc-otoferlin overexpression, leading to significant hearing improvement; the remaining three did not exhibit Myc-otoferlin transduction, resulting in smaller hearing recovery (Fig. S5). This highlights the crucial role of otoferlin overexpression in hearing restoration. In the mice with high levels of Myc-otoferlin expression, the amplitudes of ABR wave I, which reflect the electrical responses of primary auditory neurons to the sound stimulus, were 53.7% ± 24.9% (mean ± standard deviation; *n* = 5) of the mean value for *Otof*^*+/+*^ mice, showing a significantly reduced wave I amplitude despite the near normal thresholds (Fig. 1S). The amplitudes of ABR wave II were closer to the mean value for *Otof*^*+/+*^ mice (*n* = 5; mean ± standard deviation: 77.0% ± 40.5%) than were the amplitudes of ABR wave I (Fig. 1T), which may be attributed to the previously proposed central auditory compensation mechanism.^2^

In summary, we first generated the *Otof*^*p.R1934Q*/*p.R1934Q*^ mice modeling DFNB9 patients with two non-truncating alleles with a founder effect. We conducted AAV-*Otof* gene transfer in these mice to examine the possibility of hearing recovery in the context of a residual mutant otoferlin RNA/protein. We showed successful hearing recovery, with ABR thresholds approaching those of normal mice. This finding is in line with results from prior preclinical studies on truncation variants, further extending the potential of gene therapy to patients with missense variants. If translatable to humans, *OTOF* gene therapy could be successful in treating Korean and Japanese patients with DFNB9 who carry the common East Asian founder allele. The preservation of otoacoustic emission up to 5 months suggests that the therapeutic window for gene therapy in DFNB9 patients with this genotype may be longer than previously anticipated.

## CRediT authorship contribution statement

**Yehree Kim:** Writing – original draft, Visualization, Investigation, Formal analysis. **Yoojin Chung:** Writing – original draft, Visualization, Investigation, Formal analysis. **Ju Ang Kim:** Writing – original draft, Visualization, Investigation, Formal analysis. **Kyu Hee Han:** Visualization, Formal analysis. **Kwon Woo Kang:** Visualization, Investigation, Formal analysis. **Ngoc-Trinh Tran:** Visualization, Resources. **Min Young Kim:** Visualization, Investigation, Formal analysis. **Eunyoung Yi:** Visualization, Funding acquisition, Formal analysis. **Sangyong Jung:** Formal analysis, Data curation. **Bong Jik Kim:** Writing – review & editing. **Quynh-Anh Artinian:** Resources, Data curation. **Seth D. Koehler:** Resources, Data curation. **Ning Pan:** Resources, Data curation. **Tyler M. Gibson:** Resources, Data curation. **Lars Becker:** Investigation, Formal analysis. **Joseph W. Goodliffe:** Resources. **Molly Kalker:** Resources. **Madeline Barnes:** Resources. **Luke A. Shaheen:** Resources. **Meghan C. Drummond:** Resources. **Vassili Valayannopoulos:** Resources, Conceptualization. **Byung Yoon Choi:** Writing – review & editing, Supervision, Project administration, Funding acquisition, Conceptualization.

## Ethics declaration

The human study was approved by the Institutional Review Board of Seoul National University Bundang Hospital (No. B-1007-105-402). The animal study was approved by the Institutional Animal Care and Use Committee of Seoul National University Bundang Hospital (No. BA-2210-353-001), Mokpo National University (No. MNU-IACUC-2022-009, MNU-IACUC-2023-003), and Decibel Therapeutics, Inc. (Protocol 2020-011).

## Funding

This study was supported by the Basic Science Research Program through the National Research Foundation of Korea, funded by the Ministry of Education, South Korea (No. 2021R1A2C2092038 to B.Y.C.; 2022R1I1A3072036 to E.Y.), Bio Core Facility center program through the NRF (No. 2022M3A9G1014007 to B.Y.C.), and also by the Basic Research Laboratory program through the NRF, funded by the Ministry of Science & ICT (MSIT) (No. RS-2023-0021971031482092640001 to B.Y.C.) and the Technology Innovation Program (No. K_G012002572001 to B.Y.C.) funded by the Korean Ministry of Trade, Industry and Energy. This study is also funded by the Seoul National University Bundang Hospital intramural research fund (No. 16-2020-0009, 16-2022-0005, 13-2023-0002, 16-2023-0002, 18-2023-0004, and 13-2024-0004 to B.Y.C.).

## Conflict of interests

Y.C., Q.A., S.K., N.P., T.G., L.B., J.G., M.K., M.B., L.S., M.D., and V.V. are or were employees of Decibel Therapeutics or Regeneron Pharmaceuticals at the time of contribution.
