# Supplementary material for: Otof gene transfer in DFNB9 mice carrying human founder non-truncating alleles
Source: Genes Dis. 2025 Mar 6;12(5):101590. doi: 10.1016/j.gendis.2025.101590 (PMC12142502; doi:10.1016/j.gendis.2025.101590)
Supplement: Multimedia component 1 [file mmc1.docx]

**Materials and methods**

**Generation of a mouse *Otof^p.R1934Q/p.R1934Q^* model**

The founder variant of *OTOF* p.R1939Q among East Asians corresponds to p.R1934Q in the mouse *Otof* gene (GenBank accession number: NM_001100395.1; Ensembl: ENSMUSG00000062372). We therefore created a C57BL/6 mouse model with a prevalent missense p.R1934Q variant at the orthologous mouse *Otof* locus using CRISPR/Cas-mediated genome engineering. Forty-seven exons have been identified for mouse *Otof,* with the ATG start codon in exon 1 and TGA stop codon in exon 47. Codon 1934 is located on exon 47 and was therefore selected as the target site. A schematic depiction of the targeting strategy is provided in **Fig 1A**. A guide RNA (gRNA) targeting vector and donor oligo (with the targeting sequence, flanked by 120 bp homologous sequences combined on both sides) was designed as follows: gRNA1 (matches forward strand of gene): 5’CCACCCTAGCCGGCCTGACACGG; gRNA2 (matches reverse strand of gene): 5’ATGCCGTGTCAGGCCGGCTAGGG. The p.R1934Q (CGG to CAG) variant site in the donor oligo was introduced into exon 47 by homology-directed repair. A silent mutation (ACG to ACC) was also introduced to prevent the binding and re-cutting of the sequence by gRNA after homology-directed repair. The donor oligo sequence was 5’TGTATGGGTGGATGGTGCTGGTTATCTGACCAGCCCCTCCTGTCTTCTCCCCACCCTAGC**CAG**CCTGAC**ACC**GCATTCGTCTGGTTCCTGAACCCACTCAAATCTATCAAGTACCTCATCTGC. The variant sequence (CAG) is in bold, while the silent variant is bold, underlined, and in italics. The target region of mouse *Otof* locus was amplified by polymerase chain reaction (PCR) with specific primers.

*Cas9* messenger RNA (mRNA), a gRNA generated by *in vitro* transcription and donor oligo, was co-injected into fertilized eggs for *Otof*^+/^*^p.R1934Q^* knock-in mouse production. The F0 pups were genotyped by PCR amplification by following primers (mouse *Otof*-F: 5’ACCAGGGTCTGCCTCAATTCATC; mouse *Otof*-R: 5’ATGAAGTCCAGCAAGCAGGTGTCT) followed by sequence analysis to confirm that the p.R1934Q (CGG to CAG) variant was successfully introduced.

**Phenotyping of mouse *Otof^p.R1934Q/p.R1934Q^***

All experiments were approved by the animal care and use committee of Decibel Therapeutics and Seoul National University Bundang Hospital. Auditory brainstem response (ABR) and distortion product otoacoustic emissions (DPOAEs) of *Otof^+/+^* and *Otof^p.R1934Q^*^/^*^p.R1934Q^* mice were measured as previously described^1^.

**Quantification of p.R1934Q Otof mRNA and protein in untreated *Otof^+/+^* and *Otof^p.R1934Q/p.R1934Q^* mice**

Immunohistochemistry was conducted on cochlear tissues, followed by protein quantification. Cochleae from different genotypes were fixed in 4% paraformaldehyde (PFA; prepared in phosphate-buffered saline, pH 7.4), with cochlear turns being meticulously extracted. The tissues underwent incubation in a blocking/permeabilizing buffer, followed by application of primary polyclonal antibodies: otoferlin (1:100; mouse monoclonal, ab53233, Abcam, Cambridge, UK) and Vglut3 (1:200; rabbit polyclonal, 135203, Synaptic Systems, Göttingen, Germany). Post-incubation with secondary antibodies (1:400, goat anti-mouse, A11017, Invitrogen, MA, USA; 1:400, goat anti-rabbit, A21428, Molecular Probe, OR, USA), cochlear tissues were mounted using 4′,6-diamidino-2-phenylindole (DAPI) and imaged with a confocal microscope (LSM800, Zeiss, Aarhus, Denmark). Intracellular protein fluorescence was analyzed using ImageJ (version 1.54f, MD, USA), quantifying corrected total cell fluorescence.

For real-time PCR, total RNA was extracted from the entire cochlea using TRIZOL reagent (15596026, Invitrogen). This was followed by cDNA synthesis (SuperscriptIII First-Strand, 18080-51, Invitrogen). Real-time PCR was performed using the Taqman™ Fast Advanced Master Mix (4444557, Applied Biosystems, Warrington, UK), with probes Mm00453306_m1 (4331182) for otoferlin and Mm99999915_g1 (4448489) for glyceraldehyde 3-phosphate dehydrogenase (GADPH), on a QuantStudio™ 7 Flex Real-time PCR system (Applied Biosystems). The RNAscope^®^ in situ hybridization on frozen cochlear sections involved treating 5 μm thick serial sections with sequential ethanol for dehydration, followed by the RNAscope^®^ Fluorescent Multiplex Assay (Cat. No. 320851, ACDbio, CA, USA), as per the manufacturer's instructions. This utilized RNAscope Probes Mm-*Otof*-C1 (Cat. No. 485671, ACDbio) and Mm-Slc17a8-C2 (Cat. No. 431261-C2, ACDbio), with signal amplification achieved through multiple amplifiers. After counterstaining tissues with DAPI, they were visualized using a confocal microscope (LSM800, Zeiss), and mRNA expression was quantified using ImageJ, focusing on maximum intensity projections from Z-stack images.

**Quantification of inner hair cell degeneration in untreated *Otof^+/+^* and *Otof^p.R1934Q/p.R1934Q^* mice at age 4 months**

Animals were euthanized by sevoflurane overdose, and the inner ears were quickly collected. After perfusion of ice-cold 4% PFA through the oval or round window, the cochleae were post-fixed in 4% PFA for 1 h at 4°C, decalcified in 5% ethylenediaminetetraacetatic acid (EDTA) for 24 h under gentle agitation and then microdissected free of the decalcified otic capsule, the lateral wall, Reissner’s membrane, and tectorial membrane. Each cochlear preparation was cut into 2–3 pieces and was incubated in blocking/permeabilization buffer (PBS supplemented with 5% normal goal serum and 0.25% Triton X-100) for 1 h at room temperature. The tissue was then reacted with rabbit anti-myosin 6 (M5187, 1:500, Sigma, MO, USA) overnight at 4°C under gentle agitation. Following three washes with blocking/permeabilization buffer, the tissue was treated with secondary antibody (goat anti-rabbit Alexa Fluor 555^Ⓡ^, A31572, Invitrogen) for 1 h at room temperature. Excess secondary antibody was rinsed away, and the tissue was mounted on the slide using the FluorSave^Ⓡ^ mounting medium (Merck Millipore, Darmstadt, Germany). A series of images of labeled hair cells was obtained using a laser scanning confocal microscope (LSM710, Zeiss). The entire length of the organ of Corti was reconstructed in ImageJ. The number of IHCs and outer hair cells (OHCs) in each segment spanning 1% of the total length were quantified. The segments that had been damaged during tissue preparation were identified under differential interference contrast (DIC) optics and excluded from hair cell counting analysis.

**Injection of dual AAV-mOtof vectors**

*Otof^p.R1934Q^*/*^p.R1934Q^* mice aged 4.9 to 5.6 weeks (male=7, female=5) were injected with either 2 µL of dual AAV vectors (5.04 x 10^10^ vg/ear/vector of AAV1-Myo15-Myc-mOtof 5’ and AAV1-m*Otof* 3’, 1 µL each) or 2 µL of vehicle. ABR and DPOAE measurements were taken 4 weeks after treatment, after which the animals were perfused, and cochleae were harvested for wholemount histological analysis.

Animals were anesthetized using isoflurane (3% for anesthesia induction and 1.5% for maintenance). A small post-auricular incision was made using sterile scissors, and the muscles covering the posterior canal were retracted. A burr hole was made in the top of the bone of the posterior semicircular canal. The test article was mixed 9:1 with trypan blue and drawn up into the 42-gauge polyimide tubing. The polyimide tubing was sealed into the canal with surgical glue, and 2 μL of the agent was delivered at a rate of 200 nL/min. After delivery, the tubing was trimmed close to the canal and sealed shut via crimping or glue. The incision was closed with topical tissue adhesive. Animals were treated with a non-steroidal anti-inflammatory for 2 days for post-operative control of pain and were monitored for 5 days after surgery.

**Post-gene therapy auditory physiology**

For measurement of ABR and DPOAE, mice were weighed, anesthetized with ketamine/xylazine, and then placed in an acoustic chamber. Acoustic stimuli were generated on a WS4 high-performance computer workstation at a nominal sampling rate of 100 kHz, transferred to the RZ6, and delivered through a custom amplifier and dual speaker based on designs published by Eaton Peabody Labs at the Massachusetts Eye and Ear Infirmary.

**Post-gene therapy ABR data acquisition and stimulus generation**

ABRs were recorded with three needle electrodes (LifeSync Neuro, FL, USA) inserted into the skin in the dermal layers: (1) a ground electrode near the base of the tail; (2) a recording electrode at vertex along the midline of the skull between the ears; and (3) a reference electrode through the bare skin ventral to the pinna. ABR signals were filtered (100 Hz to 5000 Hz) and averaged (512 samples).

Five millisecond tone-pips with a 0.5 ms rise-fall time delivered at 81/s in alternating polarity were used for frequency-specific measurements of hearing function. Sound levels were tested up to 105 dB sound pressure level (SPL) (the equipment limit for tone-pips). Stimuli were presented in interleaved order such that a train of tone-pips containing a single presentation of each level and frequency was repeated in an interleaved ramp paradigm (as described in Buran et al. 2020)^2^.

**Post-gene therapy DPOAE data acquisition and stimulus generation**

DPOAEs were recorded with a probe tube microphone in the ear canal while the speaker delivered tones at two frequencies with a ratio of 1.2 (F2 = 1.2×F1), with the F2 level 10 dB less than that of F1. The amplitude of the distortion product (at a frequency of 2×F1 F2) was estimated using a Fast Fourier Transform algorithm. To avoid recording distortion of the acoustic system, stimulus levels for DPOAE measurement were kept below 90 dB SPL. Stimuli were presented from low to high frequencies, and from low to high levels for each frequency in sequence.

**Post-gene therapy ABR and DPOAE threshold estimation**

To estimate ABR thresholds, the signal was further filtered from 300 to 3000 Hz and the lowest sound level that elicited an ABR was selected based on visual assessment. The additional filtering resulted in a slightly different shape of the signal in absence of response between Fig 1B and 1R. The DPOAE threshold was estimated automatically by interpolating the lowest F2 sound level that elicited a response of 5 dB SPL. For both ABR and DPOAE thresholds, if no response was observed within the equipment limit it was imputed with a value 5 dB (1-step size) greater than the limit and included in the analysis.

**Immunohistochemistry**

Animals were sacrificed using CO_2_ and intracardially perfused with 10% neutral buffered formalin (NBF) following all institutional animal care and use committee (IACUC) protocol and guidelines. Temporal bones were dissected and post-fixed overnight in 10% NBF at room temperature followed by decalcification in 8% EDTA for 48 to 96 hours at room temperature. After decalcification, cochleae were dissected from temporal bones for immunohistochemistry and stained with mouse anti-Myc tag antibody (sc-40, 1:200 dilution, Santacruz, TX, USA) and rabbit anti-MYO7A (25-6790, 1:500 dilution, Proteus Bioscience, CA, USA) or rabbit anti-homer (160003, 1:800 dilution, Synaptic Systems) primary antibodies in phosphate-buffered saline solution (PBST), 0.5% Tx‑100 overnight at room temperature. Following washes, secondary antibody incubations were performed with goat anti-mouse and goat anti-rabbit antibodies (diluted 1:500 in PBST, 0.5% Tx-100) for 2 h at room temperature with DAPI nuclear counterstaining.

The presence of MYO7A positive IHCs and OHCs was evaluated and manually scored. Organ of Corti whole-organ preparations were stained using the MYO7A primary antibody, which is used to identify IHCs and OHCs based on its specificity for the endogenous MYO7A protein in the cytosol of hair cells. Cochlear frequency maps were generated using ImageJ software on organ of Corti whole-organ preparations that were imaged using the 20× objective on a Zeiss fluorescent microscope. IHCs and OHCs were counted in 200 μm × 200 μm regions of interest at half octave frequencies sampling the length of the murine organ of Corti: 8, 11.3, 16, 22.6, and 32 kHz. Hair cell numbers were determined based on the count of discrete hair cells in the evaluated 200 µm window at each position along the length of the cochlea, that is estimated according to a custom ImageJ plugin that translates cochlear position into frequency according to the published map for the mouse^3^. The total number of hair cells was defined as the sum of the number of MYO7A positive cells and the number of clearly missing hair cells in the regular pattern of the organ of Corti. Depending on the analyst and the frequency assessed, the average IHC number was between 22 and 25 cells and the number of OHCs was between 75 and 81 cells. IHCs expressing the Myc-tag were identified as cells that were co-labeled with antibodies to MYO7A and Myc and were quantified in the same manner. All hair cell counts were performed blinded to the treatment group.

**Supplementary Figures**

**
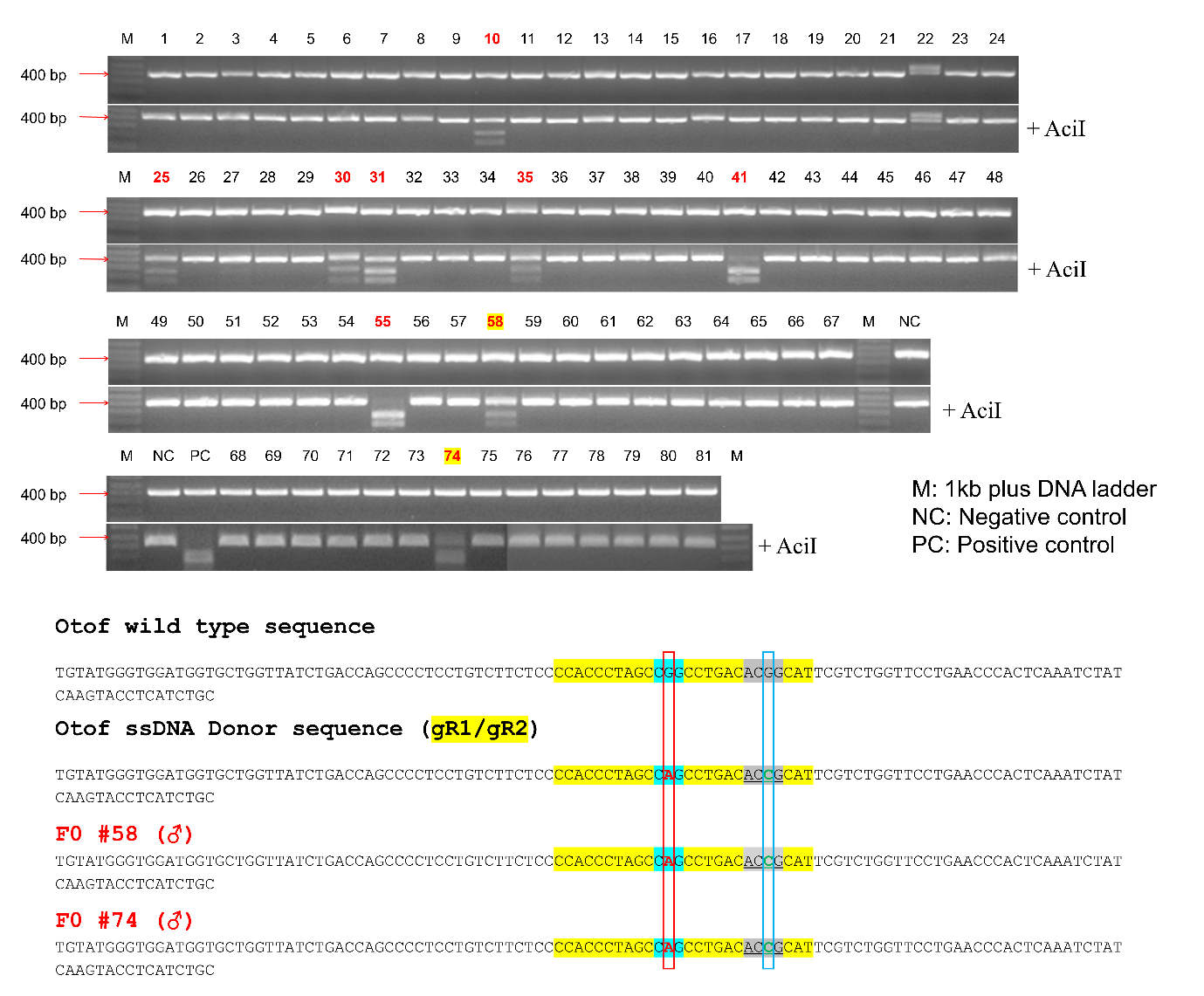
**

**Supplementary Fig. 1.** In the *mouse Otof* genome, the CGG sequence (in the blue region) was changed to CAG, resulting in the creation of  *Otof^p.R1934Q/p.R1934Q^*. To identify this mutation, a silent mutation was introduced in the ACGG sequence (in the gray region), changing it to ACCG, thereby creating an AciI recognition site. Following genotyping PCR, the amplicon was treated with AciI, resulting in the cleavage of bands into 161 bp and 246 bp fragments. This allowed for the identification of  *Otof^p.R1934Q/p.R1934Q^* F0 mice, with 9 out of 81 pups confirmed as carrying this mutation (#10, 25, 30, 31, 35, 41, 55, 58, and 74; a 10% ratio). PCR, polymerase chain reaction.

**
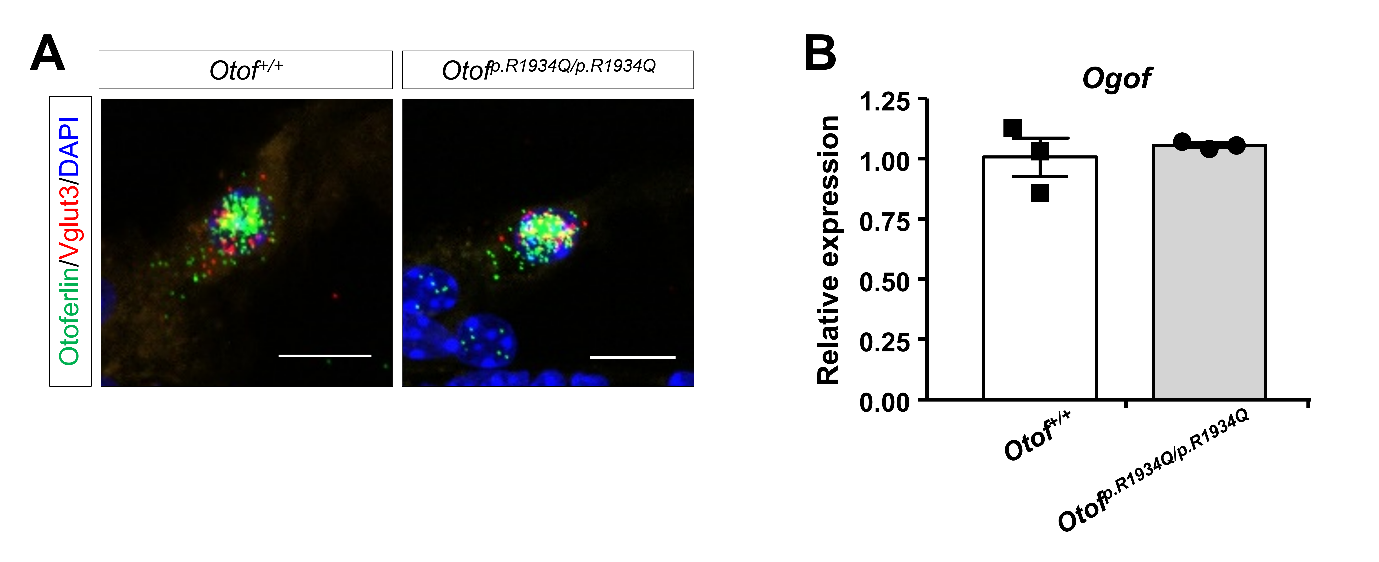
**

**Supplementary Fig. 2. mRNA expression levels in  *Otof^p.R1934Q/p.R1934Q^*.** **(A)**  *Otof* mRNA levels examined with RNAscope in situ hybridization and **(B)** real-time PCR, the gene expression level of *Otof* is similar across genotypes (n = 3 replicates, with 4 cochleae pooled per replicate). Scale bar=10 μm. DAPI, 4′,6-diamidino-2-phenylindole; mRNA, messenger RNA; PCR, polymerase chain reaction.


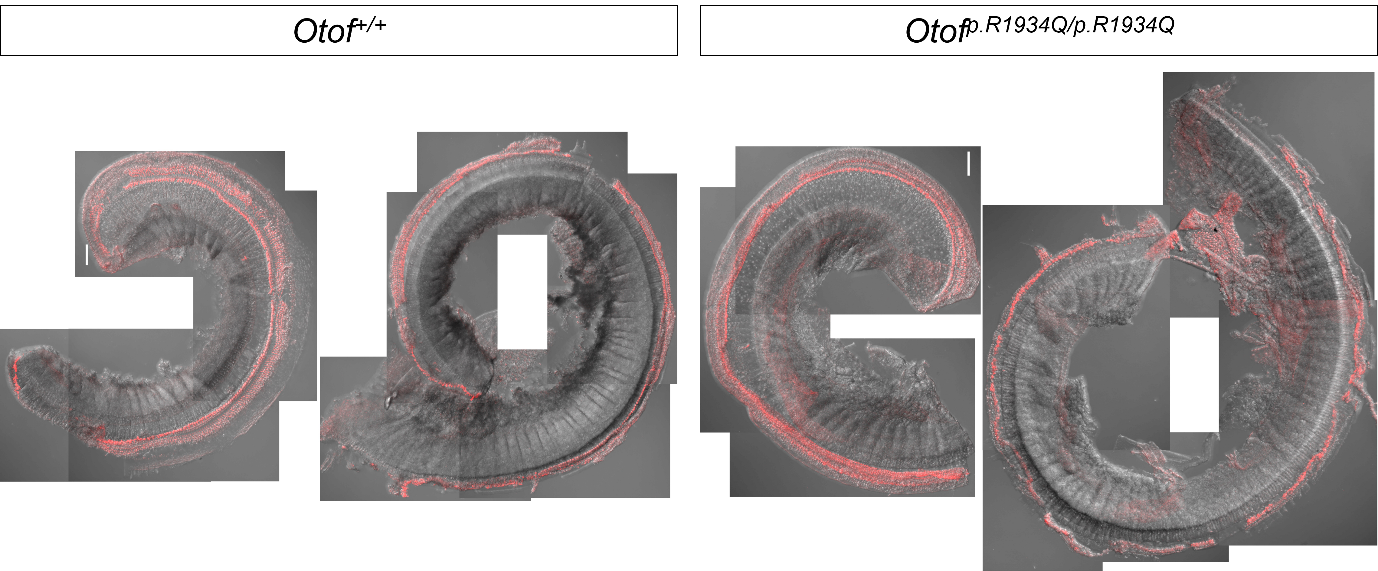


**Supplementary Fig 3. Montaged images of organ of Corti immunolabeled with anti-myosin 6.**

Fluorescence and DIC images were overlaid and reconstructed. Scale bar=50 µm.


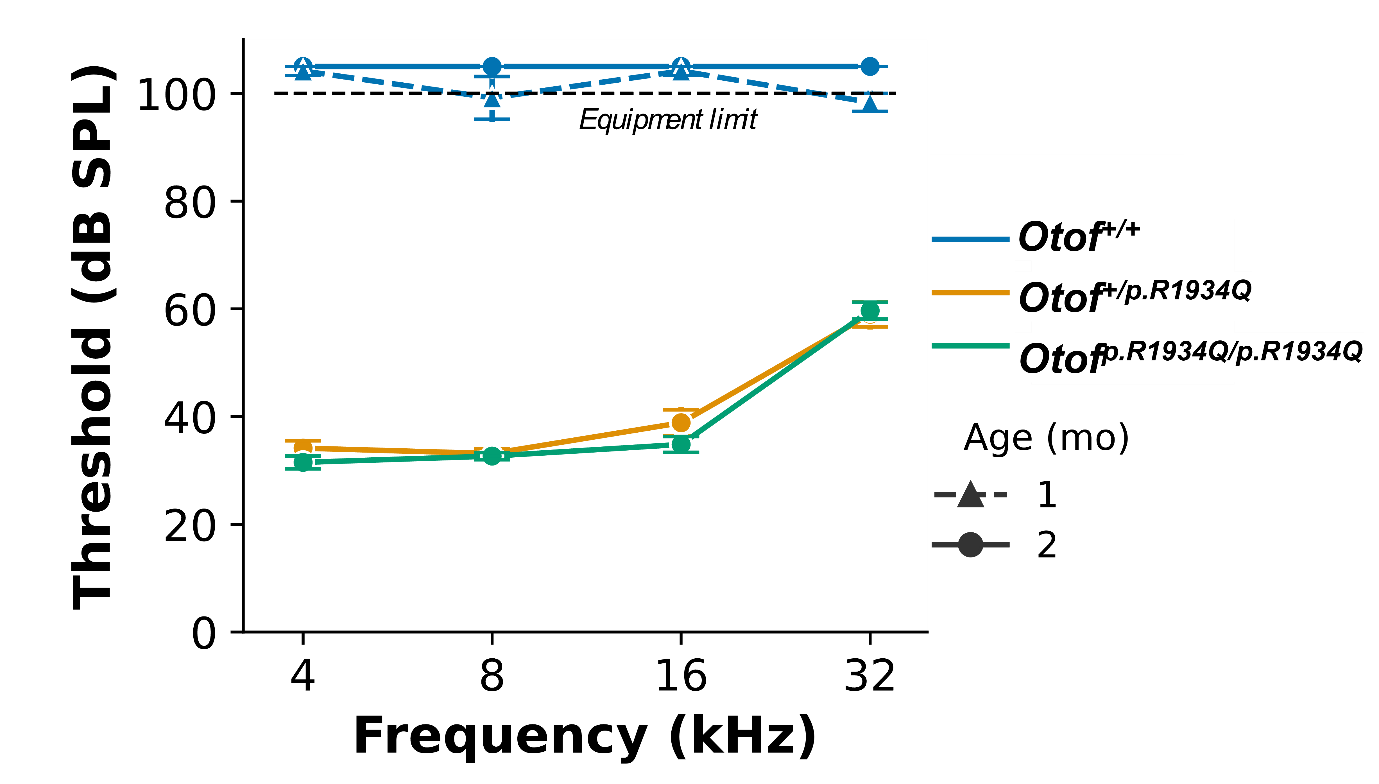


**Supplementary Fig. 4. ABR threshold of *Otof^+/+^, Otof^+/p.R1934Q^*, and *Otof^p.R1934Q/p.R1934Q^* mice.**N=30 for *Otof^+/+^*, n=35 for *Otof^+/p.R1934Q^*, and n=10 for  *Otof^p.R1934Q/p.R1934Q^* mice. *Otof^p.R1934Q/p.R1934Q^*included 1- and 2-month mice. ABR thresholds were elevated above 100 dB in all *Otof^p.R1934Q/p.R1934Q^* mice across frequencies ranging from 4 to 32 kHz. Data represented as median and interquartile range.

**
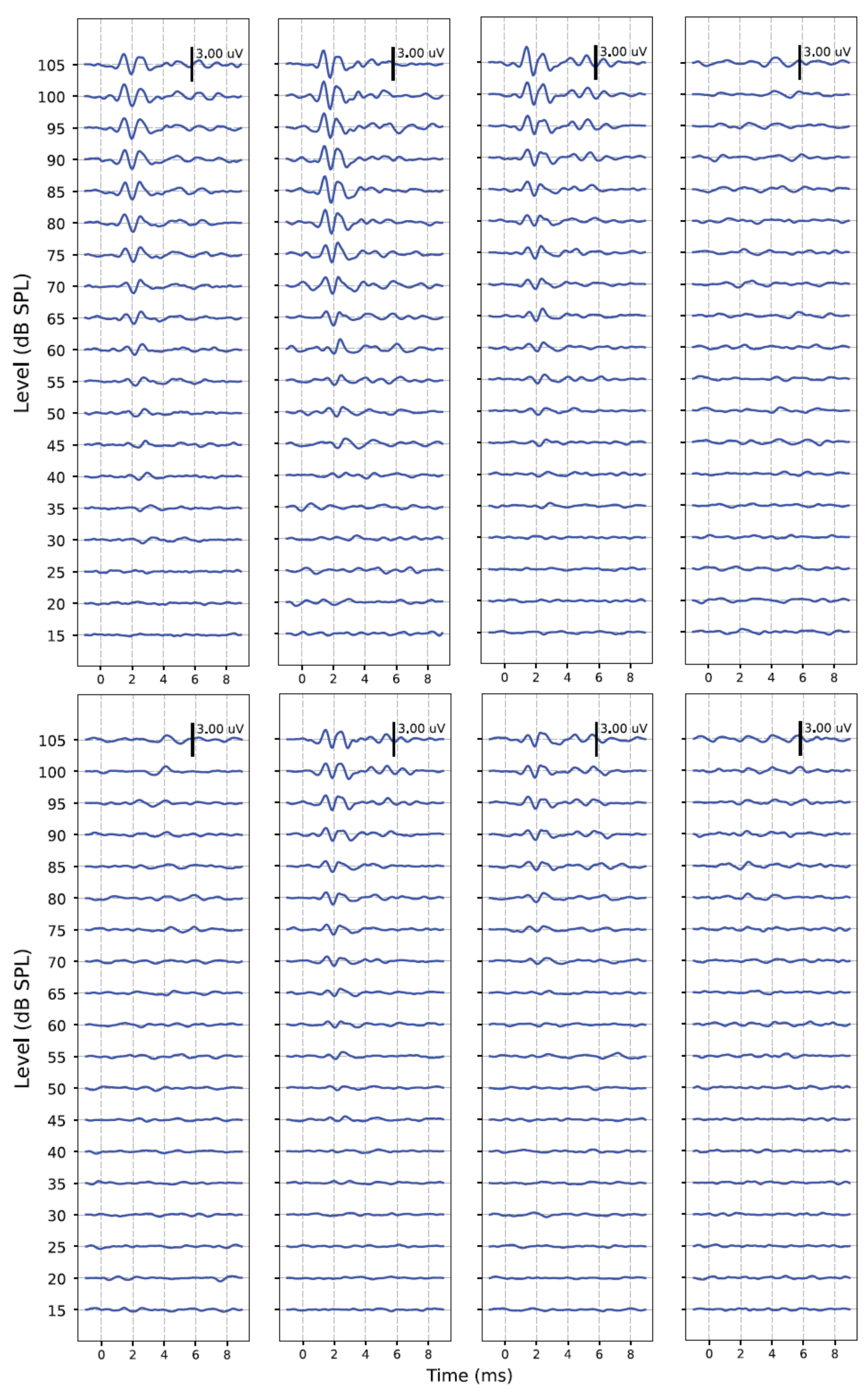
**

**Supplementary Fig. 5. ABR waveforms in response to 16 kHz tone bursts.** ABR waveforms from eight AAV-treated mice were analyzed. AAV, adeno-associated virus; ABR, auditory brainstem response.

**References**

1. Jang MW, Oh DY, Yi E, et al. A nonsense TMEM43 variant leads to disruption of connexin-linked function and autosomal dominant auditory neuropathy spectrum disorder. *Proc Natl Acad Sci U S A*. Jun 1 2021;118(22)doi:10.1073/pnas.2019681118

2. Buran BN, Elkins S, Kempton JB, Porsov EV, Brigande JV, David SV. Optimizing Auditory Brainstem Response Acquisition Using Interleaved Frequencies. *J Assoc Res Otolaryngol*. Jun 2020;21(3):225-242. doi:10.1007/s10162-020-00754-3

3. Taberner AM, Liberman MC. Response properties of single auditory nerve fibers in the mouse. *J Neurophysiol*. Jan 2005;93(1):557-69. doi:10.1152/jn.00574.2004
